# Sex and Geographic Differences in the Prevalence of Reported Childhood Motor Disability and Their Trends in Taiwan

**DOI:** 10.1155/2018/6754230

**Published:** 2018-04-05

**Authors:** Cheng-Fang Tsai, How-Ran Guo, Yen-Cheng Tseng, Der-Chung Lai

**Affiliations:** ^1^Department of Physical Medicine and Rehabilitation, Ditmanson Medical Foundation Chia-Yi Christian Hospital, 539 Zhongxiao Road, Chiayi 600, Taiwan; ^2^Graduate Institute of Clinical Medical Science, Chang Gung University, 259 Wenhua 1st Road, Taoyuan 333, Taiwan; ^3^Department of Occupational and Environmental Medicine, National Cheng Kung University Hospital, 138 Sheng-Li Road, Tainan 704, Taiwan; ^4^Department of Environmental and Occupational Health, National Cheng Kung University, 138 Sheng-Li Road, Tainan 704, Taiwan; ^5^Occupational Safety, Health and Medicine Research Center, National Cheng Kung University, 138 Sheng-Li Road, Tainan 704, Taiwan; ^6^Language Education Center and Department of Tourism, Food and Beverage Management, Chang Jung Christian University, 1 Chang-Da Road, Tainan 711, Taiwan; ^7^Department of Senior Citizen Service Management, Chia Nan University of Pharmacy & Science, 60 Erren Road, Section 1, Tainan 717, Taiwan

## Abstract

Motor disability (MD) is not uncommon in children, but data at the national level are scarce. As the Taiwan government certifies and registers disabled residents for providing services on a routine basis, the registry provides a unique opportunity for studying MD. Using data from the registry, we calculated the prevalence of MD by age, sex, and geographic area and assessed the changes from 2004 to 2010. We excluded cases under 3 years old because the government discourages the certification at this age. We found that cases between 3 and 17 years old decreased from 8187 to 6022 per year from 2004 to 2010 and the prevalence generally decreased every year in all age groups. There were more boy cases than girl cases every year, and the prevalence rate ratios ranged from 1.26 to 1.39 (*p* < 0.05 in all years), with a decreasing trend over time (*p* < 0.01). Rural areas had higher prevalence in all the years, and the prevalence rate ratio decreased from 1.31 to 1.23 (*p* < 0.05 in all years), with a decreasing trend over time (*p* < 0.05). Further studies identifying the risk factors contributing to the decreases might help in the prevention of MD in the future.

## 1. Introduction

Motor disability (MD) is major disability of children in many countries on the world [1–4]. Because MD generally lasts for the whole life, children with MD become a heavy burden to their families and societies. Many countries provide supports such as social welfare, special education, and health care to decrease the family burden [5], but these services require a lot of resources. For example, the lifetime direct and indirect costs of cerebral palsy (CP) were calculated to be 900000 USD per person [6]. Therefore, estimating the required resources precisely is important to public health, and data on prevalence can help the government to construct service plans more accurately [7].

Although MD is not uncommon in children, the reported prevalence varies widely. The variations may be attributable to factors including the differences in case definition, age range, and case-finding method [8]. For example, In China, according to the Second National Sample Survey, the prevalence of MD in children 14 years of age or younger was 0.41% in 2006 [9]. In France, a study of three birth cohorts using a registry for providing services found a prevalence of 0.334% [4]. A survey of US Census Bureau in 1997 revealed that 2.1% of children 6 to 14 years of age had difficulty in walking or running [10].

In Taiwan, according to the Disabled Welfare Act [11], the local governments are required to certify the disabled residents and provide various services, and the Ministry of Health and Welfare maintains a registry of reported cases. The Department of Statistics of Ministry of Health and Welfare [12] publishes annual summaries of the registry data, which present a rare opportunity for studying the epidemiology of MD at the national level. In a previous study, we analyzed the data and found a decreasing trend in the prevalence of MD from 2000 to 2011 [13]. To evaluate sex and geographic differences in the prevalence of MD and assess the trends of those differences and overall prevalence in Taiwanese children over time, we conducted further analyses of the data.

## 2. Materials and Methods

### 2.1. The Disability Registry System in Taiwan

The promulgation of the Disabled Welfare Act in 1980 is a milestone in the history of promoting benefits for the disable in Taiwan. A disability registry was established and covered six types of disabilities in addition to MD (visual impairment, hearing impairment or balance disability, intellectual disability, multiple disabilities, speech and language disability, and other disabilities listed by the Department of Health) initially [11]. “Multiple disabilities” means the concurrence of two or more disabilities [14]. The national disability registry of Taiwan defines MD as “motor disability that could not or hardly be repaired, caused by developmental delay, lesions of central or peripheral nervous system, traumatic, or other congenital or acquired defects or diseases of the musculoskeletal system [14].” From 1981 to 2001, nine other types of disabilities were added [15]. Local governments began to certify disabled residents and provide various services in 1980 according to the law, and patients can submit applications for certification through the local offices in their residential areas. Accordingly, the local governments report cases to the Ministry of Health and Welfare, which maintains a registry of certified cases. We have used the data from the registry to conduct a series of studies on developmental disabilities [13, 16–19].

### 2.2. Case Definition

The Taiwan Ministry of Health and Welfare registers all the cases of disability who are issued with a certificate by the local governments. Before a certificate can be issued, a patient needs to be confirmed as a case of MD by a doctor who was accredited by the government [14, 20]. According to the law, a case of MD should fit the following criteria (brief abstract, see ref. for details) [14]: (1) a joint with significant functional impairment (defined as a loss of range of motion over 70% or muscle strength of 2 or 3 on the Medical Research Council scale) in the shoulder, elbow, knee, or hip; (2) a joint with complete loss of function (defined as complete ankylosis or muscle strength of 0 or 1 on the Medical Research Council scale) in the wrist or ankle; (3) complete loss of function in the index finger and thumb of one hand, in three fingers of one hand including the thumb, or in both thumbs; (4) bilateral joints with significant functional impairment in the wrist or ankle; (5) absence of limbs at or above the level of the wrist or ankle; (6) absence of the index finger and thumb of one hand, three fingers of one hand including the index finger or thumb, five fingers or more in both hands combined, or all toes in both feet; (7) a leg length discrepancy of 5 cm (or one fifteenth) or more; and (8) abnormal tone or involuntary movement which interferes with standing or gait. Children with comorbidity of other types of disabilities (means multiple disabilities) were excluded from the analyses.

### 2.3. Data Collection

The Department of Statistics of Taiwan Ministry of Health and Welfare publishes Statistical Yearbook each year [12] (before the reorganization of the government in 2013, the reports were published by the Ministry of the Interior), and the information includes the numbers of cases by age. Whereas data on the number of cases between 3 and 17 years old by area and sex were not available in the yearbooks, we obtained the information from the Department of Statistics of Ministry of the Interior, which is available since 2004 only. On the other hand, because there was a reorganization of administration areas in 2011, we analyzed the data till 2010. Because the government discourages the certification under 3 years of age [21], we excluded cases under 3 years old from the study.

To assess the geographic differences, we defined an “urban area” as one with more than 50% of the population living in metropolitan regions, which are defined by the Directorate-General of Budget, Accounting and Statistics of Taiwan [22]. Accordingly, a “rural area” is one with 50% or less of the population living in metropolitan regions. In order to calculate the prevalence rates, we also obtained data from the Monthly Bulletin of Interior Statistics [23], which included the numbers of boys, girls, and total population by age group in area.

### 2.4. Data Analysis

We estimated prevalence rates in four age groups (3–5 years, 6–11 years, 12–14 years, and 15–17 years as categorized in the annual reports) in each year from 2004 to 2010 and evaluated the trend over the years (Table 1). The rate was estimated by dividing the number of cases by the number of individuals in a specific group. In addition, we estimated the prevalence in boys and girls in each year by dividing the number of cases by the number of individuals in each sex also from 2004 to 2010. Furthermore, we calculated the prevalence rate ratio (RR) by dividing the prevalence rate in boys by that in girls in a given year (Table 2). To evaluate the statistical significance of each RR, we calculated its 95% confidence interval (CI). In addition, we calculated the boy-to-girl ratio by dividing the number of boy cases by the number of girl cases in each year. Likewise, we estimated prevalence in rural and urban areas in each year by dividing the number of cases by the number of individuals in each type of areas from 2004 to 2010 and obtained the prevalence RR by dividing the prevalence rate in rural areas by the prevalence rate in urban areas in each year (Table 3). A 95% CI was also calculated for each RR to evaluate its statistical significance.

We presented descriptive statistics of the variables as numbers or percentages and used the chi-square test for trend to evaluate the trends of changes in prevalence. Linear regressions were used to evaluate the trends of changes in boy-to-girl and rural-to-urban RRs. We conducted all the analyses by using SAS 9.1 and performed all the statistical tests at the significance level of 0.05 (two-tailed). The study protocol was reviewed and approved by the Institution Review Board of the Ditmanson Medical Foundation Chia-Yi Christian Hospital.

## 3. Results

From 2004 to 2010, the registered cases between 3 and 17 years old constantly decreased, from 8187 to 6022 (Table 1). The prevalence decreased generally from 17.55/10,000 in 2004 to 14.89/10,000 in 2010 (*p* < 0.01). The rates generally decreased over the years in all age groups, with a few exceptions (*p* < 0.01 for all age groups). Furthermore, the prevalence rates generally increased with age in each year (*p* < 0.01 in all calendar years).

From 2004 to 2010, there were more boy cases than girl cases in each year, and the boy-to-girl ratio ranged from 1.37 to 1.51 (mean = 1.44) (Table 2). The prevalence among boys decreased from 20.10/10,000 in 2004 to 16.50/10,000 in 2010 and from 14.78/10,000 in 2004 to 13.14/10,000 in 2010 among girls. The prevalence rates generally decreased over the years in both boys and girls (*p* < 0.01). The boy-to-girl prevalence RR ranged from 1.26 to 1.39 (*p* < 0.05 in all years), with a decreasing trend over the years (*p* < 0.01).

Among the 7 cities and 18 counties in Taiwan, 7 cities and 5 counties were categorized as urban areas, and the remaining 13 counties were categorized as rural areas. From 2004 to 2010, rural areas had higher prevalence rates than urban areas. The prevalence in rural areas decreased from 21.03/10,000 in 2004 to 17.18/10,000 in 2010 and from 16.11/10,000 in 2004 to 13.98/10,000 in 2010 in urban areas. The prevalence rates generally decreased over the years in both rural and urban areas (*p* < 0.01). The rural-to-urban prevalence RRs ranged from 1.31 to 1.23 (*p* < 0.05 in all years), with a decreasing trend over time (*p* < 0.05) (Table 3).

## 4. Discussion

During the study period, the prevalence of MD in Taiwanese children decreased every year. We believe the decreasing trend was mainly attributed to the changes in criteria; that is, the qualification for receiving disability benefits may also contribute to the decreasing trend of MD. In 2006, the government published more strict criteria on the qualification for MD [24, 25] and a drop of 17.42 to 16.99 per 10000 in the prevalence was observed in 2007. For example, one of the criteria of determining disability benefits of the wrist was changed from “significant functional impairment, a joint with a loss of range of motion over 70% or muscle strength of 2 or 3 on the Medical Research Council scale [26],” to “complete functional loss, a joint with complete ankylosis or muscle strength of 0 or 1 on the Medical Research Council scale.” Because recertification after a certain period of time is required for most cases, the prevalence continued to decrease as cases went through the recertification using the more strict criteria afterwards and became disqualified. In fact, a decreasing prevalence was also observed in the MD in adults [12], which supports the effects of the changes in criteria.

In addition, decreases in the occurrence of the major causes of MD might also contribute to the decrease in the prevalence of MD. A study of three birth cohorts in France found that leading causes of MD of children were CP (0.116%, 35%) and the prevalence of CP decreased from the 1972 cohort to the 1976 cohort and then increased in the 1981 cohort, while the prevalence of MD also decreased from the 1972 cohort to the 1976 cohort and then increased in the 1981 cohort [4]. In 2004, the International Working Group on Definition and Classification of Cerebral Palsy defined CP as “a group of permanent disorders of the development of movement and posture, causing activity limitation, that are attributed to nonprogressive disturbances that occurred in the developing fetal or infant brain [27],” and the occurrence of CP has been either stable or decreasing in some developed countries over the past two decades (from late 1980 to present) [28]. While we believe the prevalence of CP children also decreased in recent years in Taiwan, as in some other developed countries, there were no data on the trend of CP over time available in Taiwan to clarify this issue [28].

Our study found that the prevalence rates of childhood MD generally increased with age. Because MD is generally not fatal and can seldom be cured in childhood, almost all the old cases survive to the next time period in the childhood, and, with new cases being included, the prevalence in childhood should increase with age [8]. A survey of US Census Bureau in 1997 also revealed increased rate with age: 1.8% of children aged 3 to 5 years had difficulty running or playing, but only 0.5% of children aged under 3 years had the difficulty [10].

Using “prevalence” and “child” combining with “motor disability” or “physical disability,” as keywords to search literature in the PubMed database, we identified nine studies on the prevalence rate of childhood MD in the general population [1, 4, 9, 29–34]. (Table 4) In comparison with studies in other countries, the prevalence of childhood MD we observed in Taiwan is relatively low. We believe the lower prevalence observed in our study can be attributed mainly to the case definition and case-finding method [8]. We adopted the data from a national registry of disabled persons who are severe enough to be qualified for special benefits, and therefore the cases were only a portion of overall cases of MD. For example, only one of the studies we retrieved reported data on severity, and the proportion of “moderate” and “severe” patients in MD cases was 47% (8/17) and 11% (2/17), respectively [30, 35]. If the situation is similar in Taiwan, the overall prevalence rate would be more than 26 per 10,000 (15 per 10,000 × 17/10 in 2010) if the certified cases are at least with “moderate” severity or 128 per 10,000 (15 per 10,000 × 17/2) if the certified cases must be with “severe” severity. As shown in Table 4, the prevalence rates reported by the other studies all fall into the range between 26 and 128 per 10,000. In addition, the Taiwan registry categorizes MD cases concomitant with other disabilities as cases of “multiple disabilities” instead of MD, and this might lead to further underestimation of the prevalence rates [14]. Furthermore, the case-finding method applied by the Taiwan registry is a passive approach, which was found to generally underestimate the prevalence rates because it does not include persons who were not reported to the administration [7]. Our review of literature also showed that studies on the basis of registry tended to report lower rates (33 per 10,000 or less) than household surveys which actively screened the study because case ascertainment through active screening is more likely to be complete than through passive receiving of reports (Table 4). However, active screening is difficult to perform in a large population. Consequently, both of the two nationwide studies identified by our literature review were conducted on a representative sample instead of the whole population [9, 33]. Therefore, to obtain estimates in Taiwan that are more comparable to those reported in the literature from other countries, a further study should (1) select a representative sample of the population, (2) apply an active screening to the sample, and (3) include cases that are not severe enough to obtain disability benefits.

In most epidemiological studies, males are at a higher risk of MD. For example, a birth cohorts study in France in 1985-1986 and 1989 found that, among children with MD, there were more boys (780) than girls (561) [4]. A sampling survey of all disabilities children under 6 years old in China in 2001 also found that the prevalence was higher in boys (1.45% versus 1.25%), which may be related to boys' higher susceptibility to injury [36]. Likewise, a survey of US Census Bureau in 1997 found that boys were more likely to have difficulty in moving arms or legs under 3 years of age (0.6% versus 0.3%), difficulty in running or playing from 3 to 5 years of age (2.0% versus 1.6%), and difficulty in walking or running from 6 to 14 years of age (2.4% versus 1.9%) [10]. In our study, we also observed more boy cases than girl cases, and boys had higher prevalence than girls in all years. A possible reason is that the central nervous system of young boys is more vulnerable to insults [37, 38].

We found that the prevalence rates of MD in rural areas were higher than those in urban areas, which is consistent with the data on MD from the Second China National Sample Survey on Disability in China in 2006 (2.46% versus 2.10% for all ages) [9]. A sampling survey of all disabilities children under 6 years old in China in 2001 also found that the prevalence was higher in rural areas (1.40% versus 1.33%) [36]. We believe that lower socioeconomic status and less accessibility to medical services in rural areas are the main factors contributing to the difference [39, 40]. The decrease in rural-to-urban prevalence RRs from 2004 to 2010 in our study probably indicates that the differences in the medical resources and the awareness of the disease between urban and rural areas were gradually diminishing. In China, on the contrary, the rural-to-urban prevalence RRs in all ages increased from 1987 to 2006, and the researchers attributed the trend to faster improvement in healthcare, higher awareness of disease and injury prevention, and better occupational safety in the urban areas, as well as the fact that most workers who immigrate from rural areas to urban areas tend to work on more risky jobs and returned to rural areas after being injured [9].

A major limitation of the current study is that we used “administrative prevalence” data, which do not include persons who did not apply or qualify for the services. In addition, we did not have information on individual patients because the government does not release such data and therefore is unable to explore related issues in greater details. Furthermore, the national disability registry identified MD patients who also had other disabilities as cases of “multiple disabilities,” which is not included in the category of MD, and so the number of cases might be underestimated [14]. Moreover, we used prevalence data instead of incidence data, which limits the identification of risk factors [41].

In comparison with previous studies, however, our study has some unique features besides the fact that it provides data at the national level, which are rarely available. This study has a large number of cases (e.g., 6022 in 2010 alone) and therefore is able to generate stable statistical estimates, which in turn can facilitate unbiased international comparisons—an important consideration in identifying the risk factors and constructing prevention strategies. In addition, all the cases were closely observed and certified by physicians, which makes the diagnosis reliable. Furthermore, the duration of data collection lasted for seven years, not just one year as in most large-scale studies, and therefore we are able to assess the time trend, which is rarely achieved in previous studies.

## 5. Conclusion

From 2004 to 2010, the prevalence of MD in Taiwanese children between 3 and 17 years of age generally decreased. The prevalence in rural areas was higher than that in urban areas, and the rural-to-urban prevalence RRs ranged from 1.23 to 1.31 (*p* < 0.05 in all years), with a decreasing trend over time (*p* < 0.05). The prevalence in boys was higher than that in girls (*p* < 0.05 in all years), and the boy-to-girl prevalence RRs ranged from 1.26 to 1.39 (*p* < 0.05 in all years), with a decreasing trend over time (*p* < 0.01). Whereas the statistics on MD at the national level that we generated from this study are rarely available, further analyses of data at the individual level and studies identifying the risk factors are desirable to help the prevention of MD in the future.

## Figures and Tables

**Table 1:** The prevalence (per 10,000 children)^a^ of motor disability by age in children 3–17 years of age.

Year	3–5 years			6–11 years			12–14 years			15–17 years			3–17 years		
	*N*	Pop.	Prev.	*N*	Pop.	Prev.	*N*	Pop.	Prev.	*N*	Pop.	Prev.	*N*	Pop.	Prev.
2004	1341	846130	15.85	3124	1887027	16.56	1774	973188	18.23	1948	957965	20.33	8187	4664310	17.55
2005	1227	809663	15.15	3058	1843489	16.59	1772	964802	18.37	1955	983879	19.87	8012	4601833	17.41
2006	1055	730819	14.44	3036	1826824	16.62	1748	968634	18.05	1978	961550	20.57	7817	4487827	17.42
2007	969	692164	14.00	2853	1759057	16.22	1670	972584	17.17	1977	971478	20.35	7469	4395283	16.99
2008	846	654179	12.93	2588	1682797	15.38	1612	968553	16.64	1859	963101	19.30	6905	4268630	16.18
2009	779	633676	12.29	2287	1587433	14.41	1639	969690	16.90	1738	967141	17.97	6443	4157940	15.50
2010	735	621318	11.83	2090	1538830	13.58	1542	912829	16.89	1655	971456	17.04	6022	4044433	14.89

^a^The prevalence (Prev.) was estimated by dividing *N* (number of cases) by Pop. (population) in each age group in each year.

**Table 2:** The prevalence and rate ratio by sex in children 3–17 years of age.

Year	Number of cases			Population		Prevalence (1/10,000)		
	Boy	Girl	Boy/Girl ratio	Boy	Girl	Boy	Girl	Rate ratio [95% CI]
2004	4883	3304	1.48	2429513	2234797	20.10	14.78	1.36 [1.30,1.42]^*∗*^
2005	4820	3192	1.51	2397970	2203863	20.10	14.48	1.39 [1.33,1.45]^*∗*^
2006	4687	3130	1.50	2339532	2148295	20.03	14.57	1.38 [1.31,1.44]^*∗*^
2007	4423	3046	1.45	2292296	2102987	19.30	14.48	1.33 [1.27,1.40]^*∗*^
2008	4030	2875	1.40	2225403	2043227	18.11	14.07	1.29 [1.23,1.35]^*∗*^
2009	3748	2695	1.39	2167065	1990875	17.30	13.54	1.28 [1.22,1.34]^*∗*^
2010	3477	2545	1.37	2107777	1936656	16.50	13.14	1.26 [1.19,1.32]^*∗*^

Boy/Girl: boy-to-girl ratio, obtained by dividing the number of boy cases by the number of girl cases in each year; CI: confidence interval; ^*∗*^*p* < 0.05.

**Table 3:** The prevalence and rate ratio by geographic area in children 3–17 years of age.

Year	Number of cases		Population		Prevalence (1/10,000)		
	Rural	Urban	Rural	Urban	Rural	Urban	Rate ratio [95% CI]
2004	2869	5318	1363979	3300331	21.03	16.11	1.31 [1.25,1.37]^*∗*^
2005	2758	5254	1339995	3261838	20.58	16.11	1.28 [1.22,1.34]^*∗*^
2006	2699	5118	1302738	3185089	20.72	16.07	1.29 [1.23,1.35]^*∗*^
2007	2564	4905	1270284	3124999	20.18	15.70	1.29 [1.23,1.35]^*∗*^
2008	2365	4540	1229992	3038638	19.23	14.94	1.29 [1.22,1.35]^*∗*^
2009	2167	4276	1195230	2962710	18.13	14.43	1.26 [1.19,1.32]^*∗*^
2010	1983	4039	1154481	2889952	17.18	13.98	1.23 [1.16,1.30]^*∗*^

CI: confidence interval; ^*∗*^*p* < 0.05.

**Table 4:** Prevalence rates of childhood motor disability in different studies.

Study	Country	Age (year)	Case definition	Case finding method	Case number (population)	Prevalence (per 10,000)
McLaren et al. (1986)	South Africa	0–10	Walking disability	Household survey and evaluations	4 (630)	63
Paul et al. (1992)	Jamaica	2–9	Gross motor disability: positive for the Ten-Question screen, followed by clinical examination (81% response rate) fitting criteria for the International Epidemiological Study on Childhood Disability	Household survey and evaluations	17 (4429)	38
Rumeau- Rouquette et al. (1992)	France	4–17	Motor disabilities: including all motor or tonicity abnormalities of any origin	Regional registry	1355 (405160)	33
Rumeau- Rouquette et al. (1997)	France	8–17	Motor disabilities: including cerebral palsy and other motor disabilities	Household survey	1309 (325347)	40
Cans et al. (2003)	France	7	Motor disabilities: including cerebral palsy and other motor disabilities	Regional registry	558 (175919)	32
Sauvey et al. (2005)	Nepal	<20	Mobility impairment	Household survey	735 (87599)	84
Luan et al. (2008)	China	0–14	Physical disability: fit the criteria listed by the China National Sample Survey on Disability	Household survey of a nationwide sample		
				1987 survey	1436 (460618)	31
				2006 survey	1960 (479581)	41
Trani et al. (2008)	Afghanistan	0–14	Physical disability: positive for the screening tool of the National Disability Survey in Afghanistan	Household survey of a nationwide sample	unavailable	0–4 years: 30 5–14 years: 80
Murthy et al. (2014)	Bangladesh	<18	Substantial physical impairment: identified by the Washington Group Questions as functional limitations in core domains and lasting for 6 months duration (or from birth)	Key informant methodology	1601 (258000)	62
				Household survey	65 (8120)	80
Our study	Taiwan	3–17	Motor disability: confirmed by a physician as fitting the criteria for receiving disability benefits	National registry		
				2004	8187 (4664310)	18
				2010	6022 (4044433)	15

## Abbreviations

CI: Confidence interval
CP: Cerebral palsy
MD: Motor disability
RR: Rate ratio.
